# Hematemesis in a Newborn: A Case Report

**DOI:** 10.7759/cureus.6784

**Published:** 2020-01-27

**Authors:** Jasmeet Kataria-Hale, Liz Febo-Rodriguez, Shweta Parmekar

**Affiliations:** 1 Pediatrics, Texas Children's Hospital, Houston, USA; 2 Pediatrics, Baylor College of Medicine, Houston, USA

**Keywords:** hematemesis, melena, newborn, eosinophils, ulcer

## Abstract

We present the case of a term-healthy neonate who developed hematemesis while being admitted at the newborn nursery. The infant was found to have gastric ulcers with duodenal eosinophils. The condition was conservatively managed and the symptoms, including ulcers, resolved with time; however, the etiology of the ulcers is still unknown.

## Introduction

Peptic ulcers and upper gastrointestinal (GI) bleeding mostly occur in critically ill patients secondary to physiologic stress [1]. The differential for GI bleeding includes medication side-effects, infection, coagulopathy, milk protein allergy, and vascular or anatomic anomalies [2]. Although primary duodenal ulcers are rare in neonates, gastric ulcers usually occur in the setting of serious underlying illness [3]. In addition to physiological stress, perinatal events that disturb GI tract circulation have been suspected in cases of gastric ulcers. We describe a case of a term-healthy neonate who developed hematemesis and melena due to primary gastric ulcers without a known inciting perinatal event.

## Case presentation

A one-day-old, East Asian, 3,890-gram term male baby, born to a 31-year-old G3P1021 mother via spontaneous vaginal delivery, developed blood-tinged emesis in the newborn nursery. The mother reported good prenatal care, negative screening labs, prenatal vitamin intake, and no tobacco, alcohol, or illicit drug use. The pregnancy had been uncomplicated. There had been no concern for or symptoms of maternal Helicobacter pylori (H. pylori) infection. She had presented in active labor with clear amniotic fluid and had had an uncomplicated delivery. APGAR scores had been 8 and 9 at one and five minutes, respectively. Vitamin K had been given at birth.

The infant had initially breastfed, though one feed of term formula had been given due to inadequate breastmilk, followed by the occurrence of blood-tinged emesis. No maternal nipple bleeding had been reported. Vital signs were within normal limits and physical exam was reassuring. On day of life (DOL) two, the infant developed melena.

A complete blood cell count showed a white blood cell count of 28 K/uL with 58% neutrophils, hemoglobin of 15 g/dL, hematocrit of 43%, and platelet count of 255 K/uL. Within 24 hours, the subsequent hematocrit was found to be 27% and packed red blood cells were transfused. Chemistries, liver panel, and coagulation studies were unremarkable. An abdominal radiograph revealed nonspecific, mildly dilated, and featureless right lower quadrant bowel loops. Blood culture showed no growth. Apt-Downey test and serum viral studies were negative. A stool panel was negative for bacteria and parasites. The gastrin level was normal.

**Figure 1:**
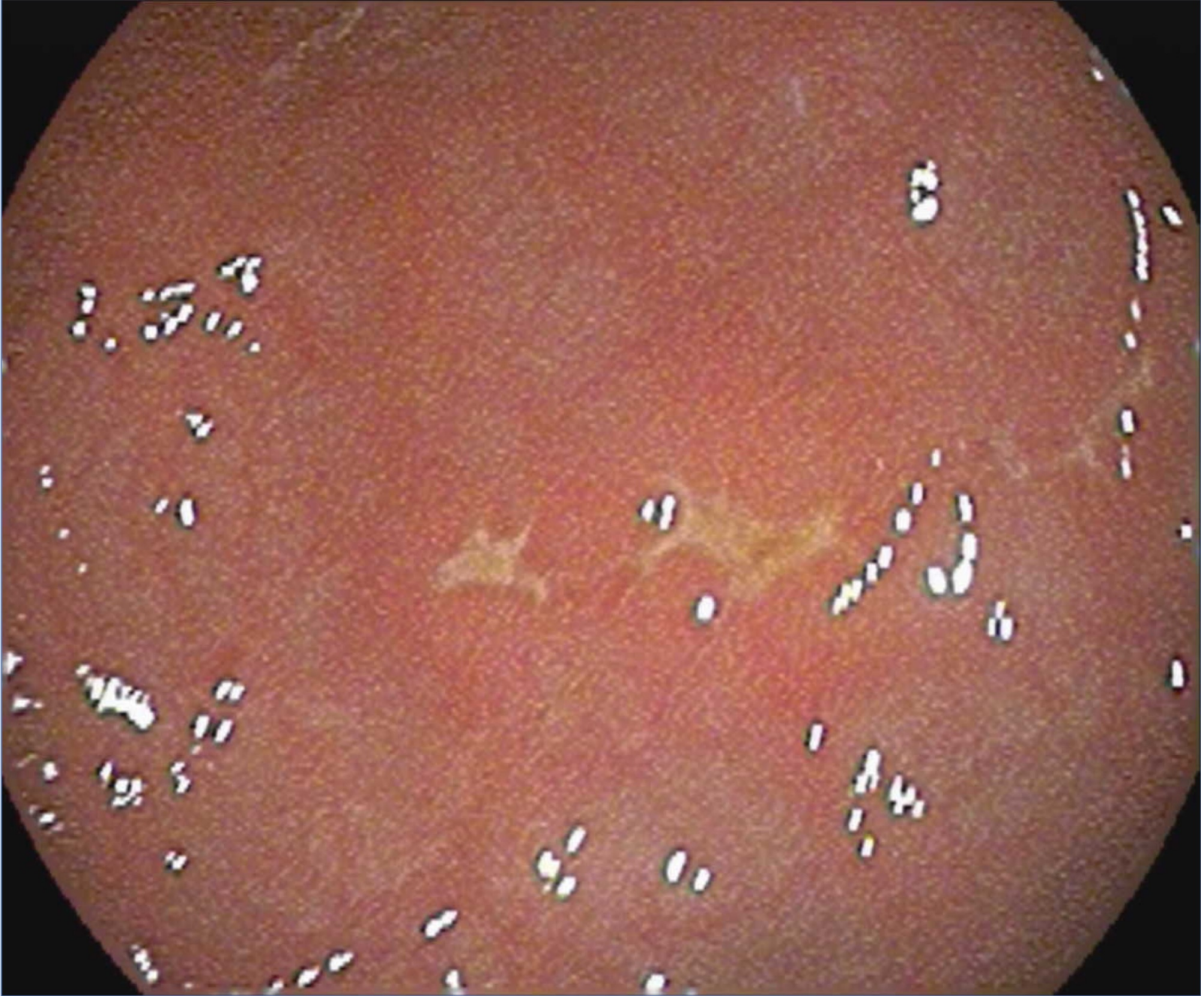
EGD showing multiple stellate gastric ulcers EGD: esophagogastroduodenoscopy

**Figure 2:**
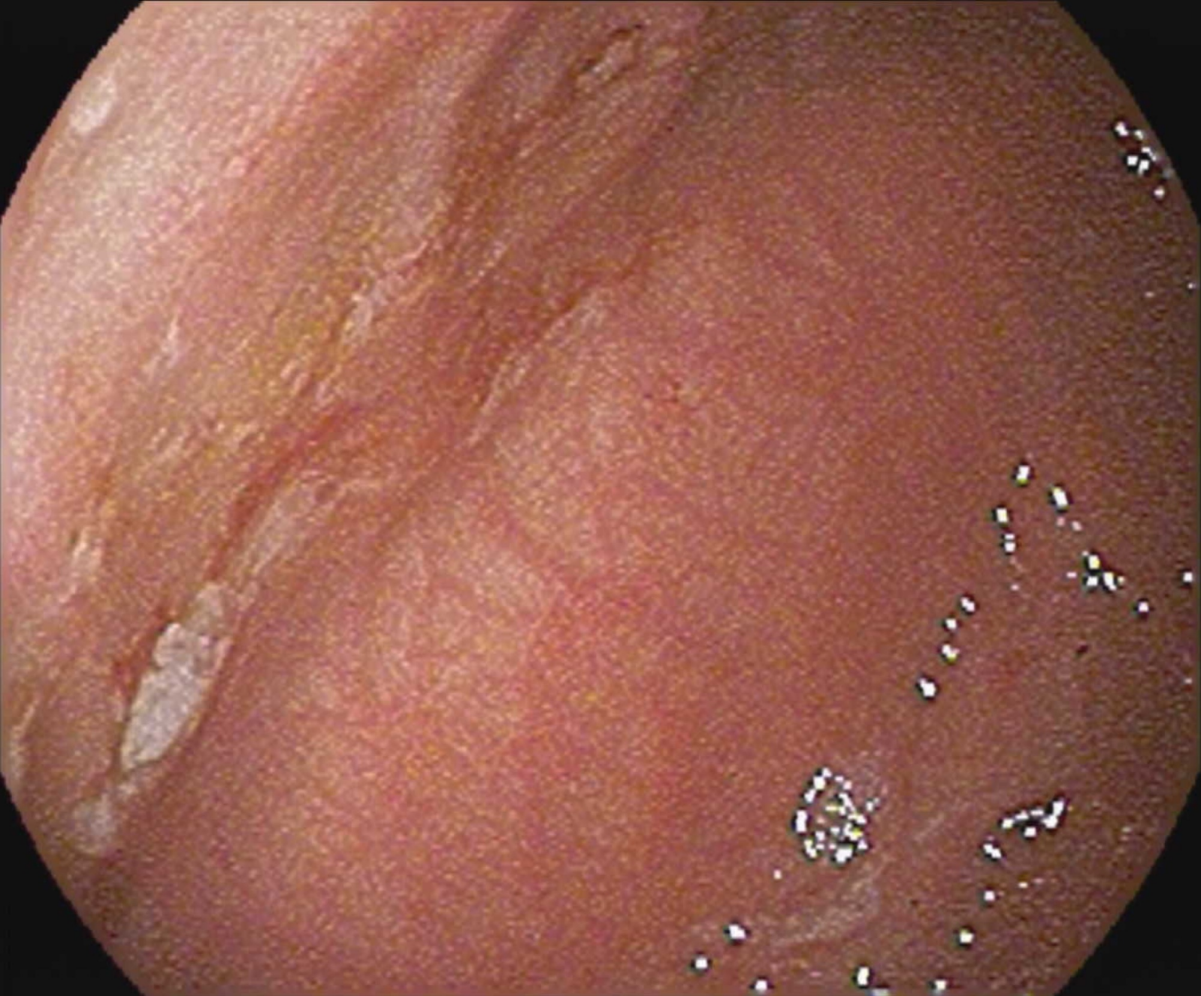
EGD showing large healing gastric ulcer that demonstrates stigmata of previous bleeding EGD: esophagogastroduodenoscopy

Initially, feeds were withheld; intravenous fluids, empiric antibiotics, and acyclovir were administered, and a nasogastric Replogle tube was placed. The gastroenterology team was consulted and an esophagogastroduodenoscopy (EGD) was performed on the fourth DOL. The EGD showed normal esophageal mucosa, extremely friable and erythematous gastric folds, multiple superficial small ulcers without active bleeding within the antral mucosa, two stellate ulcers in the gastric body, and friable and erythematous duodenal folds without ulceration (Figures 1, 2).

The duodenal biopsy was negative for H. pylori; however, it showed a focal superficial aggregation of eosinophils. Pantoprazole was empirically started. The hematemesis and melena self-resolved on the fourth DOL. The infant was discharged on the eighth DOL on full feeds and lansoprazole without recurrence of symptoms.

## Discussion

The majority of peptic ulcers in critically ill neonates are secondary to physiologic stress, such as shock, respiratory failure, sepsis, hypoglycemia, severe burns, and intracranial lesions. In addition to postnatal events, perinatal ones may contribute to peptic ulcer development in neonates. These include asphyxia, prolonged labor, instrumentation, Caesarean section, and even social stress [1,3-7]. Asphyxia as a result of circulatory disturbances of the GI tract can cause mucosal ischemia and resultant peptic ulcers. Physiological stress can impair the normal homeostasis that is maintained in the gastric mucosa, causing the formation of both superficial and deep erosions. These erosions can spread and deepen, eventually causing perforation [1]. This case is unique in that, despite a thorough work-up, a definitive etiology was unidentified; hence it indicates the need for considering the role of perinatal stress in neonatal ulcer development. 

After an uncomplicated delivery, the infant was admitted to the nursery, thereby decreasing the likelihood of postnatal physiologic stress as the cause of ulcers. Zollinger-Ellison syndrome was considered, but the gastrin level was normal. Given that melena occurred following formula intake, an immunoglobulin E-mediated cow’s milk protein allergy was considered, but the temporal relationship of symptoms after one feed and lack of hematochezia made this less likely [8]. The focal superficial aggregation of eosinophils in the duodenum on EGD is unique. In adults, eosinophils in the stomach and duodenum are thought to be a secondary response to chronic inflammation due to H. pylori infection, which was negative in this case. Another consideration was that eosinophils can accumulate in the GI tract due to food allergies, but is highly unusual in the newborn period. Despite the duodenal eosinophils, the patient did not meet diagnostic criteria for eosinophilic duodenitis and thus is an unlikely explanation for the ulcers [9,10].

Following discharge, during a retrospective inquiry, the mother revealed that she had experienced significant psychosocial stress throughout her second trimester of pregnancy. A natural disaster had caused substantial upheaval in her life, including evacuation from her home while preparing for childbirth. Moreover, she had been working two jobs with very little family support. To the best of our knowledge, there are few reports that associate gastric ulcers with psychosocial factors. However, the literature supports the concept that exposure to prenatal stress can disrupt the health and development of the fetus [11]. In this case, the psychosocial stress that the mother faced during the pregnancy may be associated with the development of ulcers. A case by Pugh et al. have described the case of an infant with fatal bleeding during the first DOL due to gastric ulceration. It was suspected to be associated with significant maternal social stress during the third trimester of pregnancy caused by maternal hormone transplacental transfer [7]. Gastrin has been previously implicated in such cases, but recent reports question the transplacental passage of this hormone to the human fetus and the role it plays in isolated ulcer development [12]. Matsueda et al. have presented a case of a stress-induced gastric ulcer in a previously healthy toddler possibly due to psychological stress [13]. The effect of perinatal stress during pregnancy on gut development in neonates has been rarely reported and further work in this area is required. At the time of this report, the infant is 19 months old with no reported GI bleeding or food allergies.

## Conclusions

This case demonstrates the potential for a healthy term infant with no known inciting factors to develop gastric ulcers. In severe or difficult cases, such as in this patient, an endoscopy may be helpful for diagnosis and potential therapeutic management. The case highlights the importance of conducting a thorough history, including prenatal maternal stressors and events, to assess possible causes for neonatal ulcers. Given the eosinophils in the duodenum, this case could represent the early findings of a food allergy; but due to the difficulties to diagnose in the newborn period, the infant would require further evaluation in childhood. This early knowledge could be predictive of future diagnoses. The etiology of the bleeding continues to be elusive and further exploration is needed, especially on the neurobiology of perinatal stress and its role in intestinal development, to help in the early identification and diagnosis of the condition.
